# Rapid Adaptation to Changing Mechanical Load by Ordered Recruitment of Identified Motor Neurons

**DOI:** 10.1523/ENEURO.0016-20.2020

**Published:** 2020-05-21

**Authors:** Jeffrey P. Gill, Hillel J. Chiel

**Affiliations:** 1 Department of Biology, Case Western Reserve University, Cleveland, Ohio 44106-7080; 2 Departments of Biology, Neurosciences, and Biomedical Engineering, Case Western Reserve University, Cleveland, Ohio 44106-7080

**Keywords:** Aplysia, feeding, force generation, identified neurons, neuromechanics, size principle

## Abstract

As they interact with their environment and encounter challenges, animals adjust their behavior on a moment-to-moment basis to maintain task fitness. This dynamic process of adaptive motor control occurs in the nervous system, but an understanding of the biomechanics of the body is essential to properly interpret the behavioral outcomes. To study how animals respond to changing task conditions, we used a model system in which the functional roles of identified neurons and the relevant biomechanics are well understood and can be studied in intact behaving animals: feeding in the marine mollusc *Aplysia*. We monitored the motor neuronal output of the feeding circuitry as intact animals fed on uniform food stimuli under unloaded and loaded conditions, and we measured the force of retraction during loaded swallows. We observed a previously undescribed pattern of force generation, which can be explained within the appropriate biomechanical context by the activity of just a few key, identified motor neurons. We show that, when encountering load, animals recruit identified retractor muscle motor neurons for longer and at higher frequency to increase retraction force duration. Our results identify a mode by which animals robustly adjust behavior to their environment, which is experimentally tractable to further mechanistic investigation.

## Significance Statement

Understanding adaptive motor control requires studying the brain and body together during behavior. Studying motor control systems at the level of individual neurons in intact animals is challenging. The *Aplysia* feeding system has individual neurons that can be identified from animal to animal and well studied biomechanics. Prior work showed that animals respond adaptively to changing mechanical load but did not measure or find strong neural correlates. As animals generate increasing force on food, we find that the increased activity and size-ordered recruitment of identified motor neurons allow animals to adapt their behavior. This is the first demonstration of a relationship between identified motor neurons and adaptive motor behavior in intact, behaving *Aplysia* in response to changing mechanical load.

## Introduction

To survive under changing environmental conditions, animals adapt their behavior on a moment-to-moment basis (Pearson, 2000). For example, as a hiker navigates rough terrain, each step requires adjustments of foot placement, stance duration, and force generation to maintain stability and to progress. As a hummingbird encounters a gust of wind, it makes rapid corrections to recover stable flight (Badger et al., 2019). Food oral processing involves instant-to-instant decision-making about chewing, bolus molding, and transport for swallowing that depends on the changing mechanical properties of food (Chen, 2009).

How can neural mechanisms that facilitate these short-term phase-dependent behavioral adaptations be investigated? Animals use sensory feedback in a closed-loop system to make these adjustments, so static analyses of components in isolation—sensory inputs, biomechanics, or the neural circuitry—may not be sufficient to understand how perturbations play through the system (Chiel and Beer, 1997; Tytell et al., 2011; Gomez-Marin and Ghazanfar, 2019). Furthermore, as intact animals engage in natural behaviors, their perceptions are modulated by the task (Niell and Stryker, 2010; Busse et al., 2017). Moreover, peripheral biomechanics must be understood to interpret motor outputs. For instance, the function of a cockroach leg muscle depends on whether the animal is stationary or moving (Sponberg et al., 2011), and the function of a molluscan feeding muscle depends on the position of other parts of the mouth (Sutton et al., 2004b).

These challenges can be overcome by studying behavioral responses to perturbations and their neural control in experimentally tractable animal models. Understanding the control of behaviors like locomotion (Grätsch et al., 2019) is advancing with the aid of powerful tools like optogenetics that target specific cell types modulating locomotion speed (Caggiano et al., 2018). Neuronal populations can be associated with behavioral functions and controlled in intact animals (Josset et al., 2018). Investigating neural mechanisms of rapid behavioral adjustments at the level of individual identified nerve cells in entirely intact, freely behaving animals is especially challenging, but is necessary for a more complete understanding of detailed circuit dynamics during behavior.

The marine mollusc *Aplysia* has been studied for many years to understand learning (Susswein et al., 1986; Chiel and Susswein, 1993; Brembs et al., 2002; Baxter and Byrne, 2006), memory (Hawkins et al., 2006), and feeding behavior (Kupfermann, 1974; Morton and Chiel, 1993a; Cropper et al., 2004) at the cellular level. Feeding in *Aplysia* has the following experimental advantages: behaviors can be reliably evoked and are controlled by a small number of large cells that can be identified repeatedly in different animals (Lu et al., 2013) during intact behavior (Cullins and Chiel, 2010).

Previous behavioral studies in *Aplysia* showed that animals adapt to load when feeding by slowing the rate of swallowing and cutting or releasing food when load becomes too great (Hurwitz and Susswein, 1992). The original studies were performed in intact animals without neural correlates. An initial study that measured neural correlates of feeding in intact animals imposed relatively small variations in load, found that load had little effect on motor recruitment and concluded that effective movements are only weakly associated with any one neural correlate (Lum et al., 2005). The much larger forces that animals generate on their own when swallowing tough foods in their natural habitat have not been measured, nor have the neural correlates of adaptation to high loads. Recent studies of the biomechanics of feeding (Neustadter et al., 2002, 2007; Kehl et al., 2019), modeling (Neustadter et al., 2002; Sutton et al., 2004a), and neural control (McManus et al., 2014; Lu et al., 2015; Cullins et al., 2015b) have provided further insights into the behavioral roles of individual neurons, suggesting that it might now be possible to relate behavior to neural activity despite the high variability.

In the present study, we report behavioral and neural responses at the level of identified neurons in intact animals to a well defined mechanical load that elicits an adaptive change in behavior. Motor neurons are recruited in size order, and these recruitment changes can be interpreted through an understanding of the biomechanics. Our results suggest that, despite neural variability, clear relationships can be defined between specific neurons and phases of force generation in response to load.

## Materials and Methods

### 

#### Animal selection

Wild-caught *Aplysia californica* were acquired from South Coast Bio-Marine and kept in 151 L aquaria maintained at 16 ± 1°C with a 12 h light/dark cycle. Before selection for surgery, animal health was assessed by inspection, and readiness to feed was tested with small pieces of dried nori (Deluxe Sushi-Nori, nagai roasted seaweed, Nagai Nori USA) or dulse (Wild Dulse Premium Raw, Natural Zing), with interbite intervals of 3–5 s considered suitable (Kupfermann, 1974). Animals with masses of 200–450 g were used for this study.

#### Electrode preparation

Multichannel hook electrode assemblies were prepared using methods similar to those used in the study by Cullins and Chiel (2010). Briefly, pairs of fine wires (25 μm diameter, stainless steel 316, heavy polyimide insulated; California Fine Wire), ∼40 cm in length, were twisted together to form one single-channel differential electrode with signal and reference wires; multiple twisted pairs were twisted together to create a multichannel electrode assembly. The bundle of wires was covered in a thin coat of household silicone glue (Silicone 2+, General Electric) and allowed to dry overnight. Insulation was scraped away to expose the steel wire on both ends of each wire. Gold pins (catalog #SA3148/1, Bulgin) were soldered onto one end of each wire, and laboratory tape was used to secure the pins. The other end of the wire was prepared by shaping it into a hook for recording from nerve or muscle or into a straight segment to serve as a reference electrode. Twisted pairs were color coded with flecks of glitter and colored tape to keep track of which wire ends corresponded to which pins, and wire polarity was indicated with markings on the tape. A small ball of silicone glue was added ∼2 cm from the implantable end of the assembly to serve as an anchor inside the animal and to provide strain relief.

#### Surgery

Each animal was initially anesthetized using chilled isotonic (333 mm) magnesium chloride solution injected through the body wall into the body cavity. A volume equivalent to 15–40% of the mass of the animal was used. After a few minutes, the animal relaxed. It was then transferred to a bucket of iced artificial sea water (ASW) maintained at 1–5°C. After 10 min of complete immersion, the animal was moved to a surgical tray (∼12 cm tall, 22 cm long, and 13 cm wide) partially filled with wet sand (to a height of ∼6 cm) soaked in a small amount of ASW that was frozen before the surgery. The sand helped to maintain a low temperature throughout the surgery and prevented the animal from sinking as the ice melted. The animal rested on a cushion of filter floss to prevent direct contact with the frozen ASW/sand mixture, which could cause tissue damage. Chilled ASW was added on top to a level just high enough to keep the animal immersed, but low enough to permit the surgery. With the periodic addition of crushed ASW ice cubes and the removal of warmed water, the bath was kept at 1–5°C throughout the surgery. At this temperature, the animal remained anesthetized and relaxed for many hours. An aquarium air stone was placed near the gills to prevent hypoxia.

Electrode implantation methods used were similar to those described by Cullins and Chiel (2010), with a few modifications. Briefly, the animal was positioned dorsal side up and tilted on its right side; surgery was always performed on the left side to avoid the dextrally lateralized reproductive organs. A loop of stiff wire mounted on a manipulator was positioned beneath the head and was used throughout the surgery to stabilize, elevate, and reposition the buccal mass. Using a scalpel, a 1-cm-long incision was made below the eye spot parallel to the anterior–posterior body axis to gain access to the buccal mass. The incision was held open using retractors mounted to the surgical tray, which elevated the incision and prevented the escape of hemolymph and the introduction of the bath fluid into the body cavity. Hook electrodes from the multichannel electrode assembly were attached to a band of the I2 protractor muscle, the radular nerve (RN), buccal nerve 2 (BN2), and BN3 (nerve nomenclature is based on the study by Gardner, 1971; but also see Scott et al., 1991). As in the study by Cullins and Chiel (2010), Kwik-Sil biocompatible silicone adhesive (World Precision Instruments) was used to insulate the recording electrode wires; unlike in the study by Cullins and Chiel (2010), Super Glue was not first used to secure the wires, as Kwik-Sil was sufficient for this purpose. At the low temperatures maintained throughout surgery, Kwik-Sil took much longer to cure than it would at room temperature. This extended the duration of the surgeries, but the advantage of longer working times under ice anesthesia outweighed this drawback.

After electrode attachment, the incision was closed with a silk suture, and the animal was returned to the main aquarium and housed in an isolated tank to recover for 1–3 d, after which it was again willing to feed.

#### Experimental setup

Animals were moved into a 1.8 L cylindrical (15 cm diameter) perforated plastic containment tank positioned inside a larger 8.5 L rectangular reservoir tank of ASW maintained at 16 ± 1°C. The bath was aerated by an air stone. To encourage feeding, nonsurgical animals were sometimes placed in the reservoir tank and fed, as the presence of conspecifics is known to increase food arousal in a related *Aplysia* species, likely via pheromone release (Ziv et al., 1991). Contact between the primary subject and the other animals was prevented by a perforated barrier.

Electrode signals were amplified and filtered by A-M Systems Model 1700 Differential AC Amplifiers. Nerve signals (RN, BN2, BN3) were filtered with a 300 Hz low cutoff and a 500 Hz high cutoff; muscle signals (I2) were filtered with a 10 Hz low cutoff and a 500 Hz high cutoff. All signals were digitized at 5000 Hz using a digitizer (catalog #PCIe-6251 and #BNC-2111, National Instruments) and recorded using AxoGraph X (AxoGraph Scientific) in continuous acquisition mode.

A camera (HD Pro Webcam C920, Logitech) was positioned over the containment tank for recording behavior at 30 frames/s throughout the experiment. A digital counter (model C342-0562 totalizing counter, Veeder-Root) was programmed to increment at 10 Hz when the recording software was started; the counter was visible in the simultaneous video recording at the start and end of the experiment, and pulses to the counter were recorded in AxoGraph X so that video and signals could be synchronized later.

A force transducer (model FT03E, Grass Instrument Division, Astro-Med) was mounted above the containment tank. The force transducer was calibrated for isometric measurements up to at least 1000 mN, with red/black multiplying springs installed to increase its maximum working range up to 2000 mN. Force levels were amplified and filtered (200 Hz high cutoff) by a strain gauge conditioner (model 3170, Daytronic) and digitized at 5000 Hz with the National Instruments hardware. The force measurement was recorded in AxoGraph X simultaneously with the electrodes and digital counter pulses.

Animals were unconstrained within the containment tank during feeding to encourage natural behavior. To elicit biting behavior, animals were presented with dried nori prepared as uniform strips. When the animal was successful in grasping food, it immediately transitioned from biting to swallowing. Unloaded swallows were obtained when animals fed on 5 × 0.5 cm strips of dried nori. Loaded swallows were obtained when animals fed on10 × 0.25 cm strips of “unbreakable” seaweed, which were composed of two layers of dried nori reinforced with double-sided tape between them to prevent the strip from breaking under tension. Compared with the unbreakable seaweed strips, wider dimensions were used for the unloaded dried nori because it was more fragile and prone to breaking; shorter dimensions were used so that the unloaded strips would not bunch up in the buccal cavity and induce internal load. Unbreakable seaweed strips were anchored at one end to the force transducer with an alligator clip and suspended vertically over the containment tank. When animals fed on this stimulus, they tended to orient their heads toward the surface of the water directly below the force transducer; consequently, force applied by the animal on the seaweed was almost entirely downward, allowing the vertically oriented and fixed force transducer to accurately measure it. In most experiments, seaweed strips were marked at 1 cm intervals using a silver marker. Animals typically persisted in trying to swallow the unbreakable anchored strips for several minutes. This behavior was similar to that seen when animals feed on tough pieces of natural seaweed; similar behavior was seen by Hurwitz and Susswein (1992) when *Aplysia oculifera* attempted to swallow weighted seaweed strips.

Recording sessions were repeated daily as long as animals were willing to feed and the signal quality of the implanted electrodes had not severely degraded. Data used in the present study were obtained 3–5 d after surgery.

#### Data analysis

**Figure 1.**
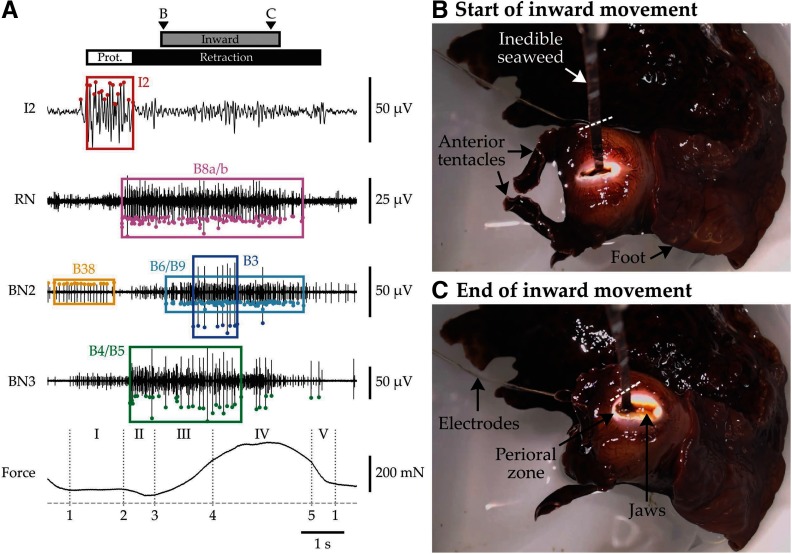
Example signals, unit identification, force segmentation, and synchronized video frames. ***A***, The first four traces show the electrical activity recorded on hook electrodes attached to the I2 protractor muscle, RN, BN2, and BN3. The last trace shows the force measured by the force transducer as the animal attempted to swallow an unbreakable seaweed strip (zero force indicated by dashed line). Colored points mark spikes detected using window discriminators. Colored boxes span each unit burst detected using frequency thresholds. Dotted vertical lines indicate events in the force record used for segmentation; Arabic and Roman numbers associated with segmentation are explained in the Materials and Methods. White and black bars above the traces indicate the protraction phase (spanning the I2 burst) and retraction phase (spanning the end of the I2 burst to the end of B43 motor neuron activity, identified as a small unit on BN2; Lu et al., 2013), respectively. The gray bar indicates the period during which the animal was pulling seaweed inward. Arrowheads labeled “B” and “C” mark video frame times. ***B***, ***C***, As the animal swallowed, it applied downward force on the unbreakable seaweed strip, which was attached to a force transducer by a clip, the end of which is just visible at the top of each frame. Seaweed strips were marked at 1 cm intervals using a silver marker; one mark is highlighted with a white dashed line in both panels to emphasize how much the animal swallowed. The wires of the electrode assembly can be seen exiting the frame at the top left. In ***B***, the grasper is fully protracted, has begun to close, and is just beginning to generate downward force. In ***C***, the grasper is fully retracted, and force is at its maximum. Important anatomic structures of the animal are also indicated with arrows.

Analysis was facilitated by the development of an open source Python application for reviewing the synchronized electrode, force, and video recordings (Gill et al., 2020). A data subset viewable with the application is shown in Figure 1, with additional annotations following from the analysis procedures described below.

*Swallow selection.* Unloaded swallows were selected for analysis from sequences in which the animal had grasped the food but in which the strip had not yet been fully ingested (i.e., while it was still visible; typically, the first three to five motor programs following initial grasping of the strip). The first ingestive behavior in which the animal initially grasped the food was not included (i.e., the initial bite-swallow; Weiss et al., 1986). Loaded swallows were selected for analysis from bouts of swallows on anchored, unbreakable seaweed only after tension had fully developed in the strip (such that force could be measured accurately; this was after 4–6 cm of the 10 cm strip had been swallowed) and before the animal began to vary its feeding strategy by interjecting long pauses between swallows, or by switching to a rasping or scraping behavior (typically, three to seven motor programs after tension had reached its maximum). The distinction between swallowing and rasping or scraping was determined by careful analysis of the video and the observation that, in swallowing, the radula of the animal did not slide relative to the seaweed strip during retraction when the grasper was closed, and the nori on the surface of the tape was not removed.

Because animals were unrestrained when feeding, there was some variability in posture when they engaged with the anchored seaweed. In sequences where the head and body of the animal were stationary because the anterior portion of its foot was well anchored to the tank, we could be confident that the measured force was generated almost purely by the buccal mass. These force records were similar to others in which the animal had anchored itself less securely to the tank using the posterior portion of its foot, which allowed its head and part of its body to move in response to the force it exerted. Under the latter conditions, some fraction of the force generated could have been exerted by the body itself, and certainly some small amount was generated by the weight of the animal if it began to lift its head out of the water due to its swallowing movements. However, because the head of the animal always advanced along the taut strip in the retraction phase when force was increasing, it was evident that the buccal mass was pulling the animal forward, rather than the animal pulling back primarily with its body. Furthermore, we did not observe the animal actively moving its body around or shortening its foot during the sequences we analyzed. The similarities between force records from well anchored and less well anchored animals suggested they could be analyzed together, so we did not distinguish them in our analysis.

One animal was strong enough to move the containment tank that it had attached its foot to during the retraction phase of some swallows. In subsequent experiments, the tank was anchored securely to prevent movement. The behavior of this animal and the force records obtained from it were similar to those of other animals, so we did not distinguish them in our analysis.

*Inward movement of seaweed.* Using the simultaneously captured video (Fig. 1*B*,*C*), the periods associated with retraction of the grasper during which the animals were moving the seaweed strips into the buccal cavity were noted and used for subsequent analyses. If the entire period of movement could not be seen due to the animal finishing the strip or turning away from the camera, the swallow was excluded from analyses dependent on this measure. If the strip appeared to stop moving inward early in the retraction phase, which may happen if a breakable strip tears internal to the buccal mass, the swallow was likewise excluded as it did not accurately correspond to the full period of force generation.

When pulling on anchored seaweed, animals typically applied force for an extended period of time before partially releasing the seaweed, at which time the seaweed would move outward. The noted periods of inward movement included these periods of high force, during which the anchored seaweed may have moved inward only a little, and ended with the reversal of seaweed movement.

*Spike detection.* As a first step toward locating the bursts of identified motor neurons and their firing frequencies, individual spikes were detected and grouped into spike trains using manually specified window discriminators, which captured signal peaks on a given channel occurring within a given amplitude range and time window. Individual motor neurons can be identified from extracellular nerve signals in this system because of their reliable relative amplitudes and phase of activity (Morton and Chiel, 1993b; Lu et al., 2013, 2015; McManus et al., 2014; Cullins et al., 2015b; Table 1). The spike amplitude of some neurons, especially B8a/b and B4/B5, can vary when these neurons activate at high frequencies due to spike collisions seen in the extracellular nerve recordings (Morton and Chiel, 1993b; Warman and Chiel, 1995), so we designed the window discriminators to account for this variability. Sources of animal-to-animal variability, such as electrode attachment site, affected the absolute amplitude of spikes, so the amplitude limits of window discriminators had to be set manually for each recording; behavior-to-behavior variability also required that burst timing was first estimated manually, so that each window discriminator was constrained to an appropriate time range, which excluded spikes with similar amplitude but incorrect timing that were likely produced by different neurons. This same procedure was applied to detecting major EMG activity in the I2 muscle record, which corresponds to the activity of protractor motor neurons B31/B32 and B61/B62 (Hurwitz et al., 1996). A 100 Hz low-pass filter was digitally applied to the I2 signal first to make peak detection more reliable. Retraction phase activity recorded on the I2 channel was excluded from the analysis because it was likely associated with the activation of the large nearby I4 muscle instead of I2 (Hurwitz et al., 1996).

**Table 1:** Criteria for unit and burst identification

Unit	Channel	Relative amplitude by channel	Timing	Start frequency (Hz)	End frequency (Hz)
B38	BN2	Medium, largest in protraction	Protraction	8	5
I2 (B31/B32, B61/B62)	I2	Largest	Protraction	10	5
B8a/b	RN	Largest	Retraction	3	3
B6/B9	BN2	Medium, 2nd largest in retraction	Retraction	10	5
B3	BN2	Largest overall	Retraction	8	2
B4/B5	BN3	Largest	Retraction	3	3

Frequency thresholds for burst initiation and termination were based on prior physiological studies and analyses (Morton and Chiel, 1993a; Hurwitz et al., 1996; McManus et al., 2014; Lu et al., 2015; Cullins et al., 2015b). These values were tried for all animals first and used if they yielded distinct bursts. In a few cases, exceptions had to be made in which frequencies were lowered for specific units in specific animals that tended to have unusually low firing rates. Named pairs of neurons (B8a/b, B6/B9, and B4/B5) are pairs of neurons that are difficult to distinguish from extracellular nerve recordings alone but are similar enough in their function to group together.

*Burst identification using frequency thresholds.* Previous work has shown that muscles in this system act as low-pass filters, and motor neuron activity may not result in measurable force if firing rates are too slow or activity is too brief (Yu et al., 1999; Lu et al., 2015). To address this, frequency thresholds were applied to the spike trains identified in the previous step to determine the start and end of candidate bursts (Table 1). For each spike train, the instantaneous firing frequency (IFF) was calculated by inverting the interspike interval. A candidate burst was identified as beginning when the IFF first crossed the start frequency threshold, and as ending when it later dropped below the end frequency threshold. Candidate bursts were discarded if they were <0.5 s or contained fewer than three spikes. This procedure often resulted in one remaining candidate burst spanning the activity of the unit. However, sometimes the unit fired more sporadically, and a set of candidate bursts was identified, each separated by a short period of low activity. Because force likely could have been maintained throughout the entire period after the first burst initiated, these candidate bursts were merged into a single burst spanning the entire set of spikes.

In some analyses, the B3 and B6/B9 motor neurons were combined due to their functional similarity (Lu et al., 2015) and are referred to collectively as B3/B6/B9. The combined unit was said to be bursting if either B3 or B6/B9 (or both) was bursting. Because the B3 and B6/B9 burst periods always overlapped at least partially, the B3/B6/B9 burst period was equivalent to the period from the start of either the B3 burst or the B6/B9 burst (whichever came first) to the end of either the B3 burst or the B6/B9 burst (whichever came last).

*Mean burst firing rate.* The mean firing rate during bursting for a unit was calculated as the number of spikes in the burst minus 1 divided by the duration of the burst.

*Smoothed instantaneous firing rate.* To create a smoothed representation of the instantaneous firing frequency for each unit, spike trains were convolved with a Gaussian kernel centered on each spike with SD σ = 200 ms.

*Force segmentation of loaded swallows.* For each loaded swallow, the timing of certain events was identified from the force record (Fig. 1*A*; events are marked with dotted lines), as follows: (1) the end of rapid force decrease in the preceding swallow, when force reached a temporarily stable intermediate value, typically around the start of protraction; (2) the start of the final drop in force to its minimum value, which occurred at the end of protraction; (3) the start of rapid force increase at the beginning of retraction; (4) the start of force deceleration as force approached its maximum value, about halfway through retraction; and (5) the start of rapid force decrease at the end of retraction. These events were identified manually from the force record, without reference to the neural record, but with regular reference to the behavior recorded in the synchronized video.

We refer to the periods between these events as I, the “partial force maintenance” phase, between events 1 and 2; II, the “force dip” phase, between events 2 and 3; III, the “force rise” phase, between events 3 and 4; IV, the “force maintenance” phase, between events 4 and 5, when force was near its maximum value; and V, the “major force drop,” which spanned events 5 and 1 in the next swallow.

In some swallows, the force did not temporarily remain at an intermediate value after the rapid force decrease of the preceding swallow, and instead the force dropped to its minimum value relatively smoothly. For these swallows, events 1 and 2 were not distinguished, and the partial force maintenance phase (I) that would occur between them was undefined.

*Time normalization.* For some aggregate analyses of all swallows from all animals, a time normalization procedure was applied to burst start and stop times, force time series, and smoothed instantaneous firing rate time series so that swallows with different phase durations could be compared with one another.

Because force records were not available for them, unloaded swallows were normalized in time by aligning the start and end of each period of inward movement, and time was rescaled uniformly. For example, to match it to another swallow with inward movement lasting 1 s, a swallow with inward movement lasting 2 s would have its total duration halved, and the two swallows would be aligned at both the start and end of inward movement.

Loaded swallows were normalized in time using the stages of force segmentation. Each stage was linearly “stretched” or “compressed” so that all swallows could be aligned at each boundary between stages. For example, to match a swallow with force segmentation stage durations (in seconds) 1, 2, 2, 1, and 1 to a second swallow with stage durations 1, 1, 2, 3, and 1, the duration of stage II of the first swallow would be halved and the duration of stage IV would be tripled, and the two swallows would then be aligned at the boundaries between phases.

Following time normalization, aggregate statistics (median and quartiles) of burst start and stop times, force time series, and firing rate time series could be computed. So that these new, statistically computed time series could be plotted with typical durations, each phase (inward movement for unloaded swallows or force segmentation stages for loaded swallows) was “stretched” or “compressed” to have the median duration observed in all swallows. This allowed the new time series for unloaded swallows to be compared directly to those for loaded swallows.

For loaded swallows with undefined partial force maintenance phases (7 of 39 swallows), time normalization was not possible in the early part of the swallow before the start of the rise phase due to a missing boundary. These portions of the time series were dropped from the aggregate analysis, as were the timings of any bursts that occurred in this period.

#### Experimental design and statistical analysis

When statistically analyzing differences in swallow cycle duration under unloaded and loaded conditions, we used a paired-samples one-tailed *t* test, with *p* < 0.05 considered significant [sensitivity analysis: with *n* = 5 animals and power 1 − β = 0.8, a standard mean difference of (Cohen’s) dz = 1.36 would be detectable at the α = 0.05 significance level; dz was calculated by the program G*Power (Faul et al., 2007), which was used for all power calculations]. After obtaining a significant result, we divided cycle duration into the following two components: durations of inward movement of seaweed; and durations between inward movements. For each, we used a paired-samples one-tailed *t* test, with *p* < 0.05/2 = 0.025 considered significant (sensitivity analysis: with *n* = 5 animals and power 1 – β = 0.8, a standard mean difference of dz = 1.68 would be detectable at the α = 0.025 significance level). Test results are reported in Table 2, along with effect sizes (Cohen’s d; Sheskin, 2011).

**Table 2:** Statistical details for changes in behavioral durations under unloaded and loaded swallowing conditions

Measure (Figure)	Animal	Unloaded	Loaded	Difference	Statistical test results
Total cycle time (Figure 2*C*)	1	5.45 ± 0.50 (4)	6.91 ± 0.07 (4)	1.65 ± 0.41 (5) 32 ± 9% (5)	Shapiro–Wilk W = 0.93, *p* = 0.58 (n.s.) Paired *t* test *t*_(4)_ = 4.078, *p* = 0.008 (sig.) Cohen's d = 2.45
	2	5.97 ± 0.58 (4)	7.48 ± 0.31 (12)		
	3	4.76 ± 0.28 (4)	7.80 ± 0.64 (4)		
	4	4.65 ± 0.16 (3)	6.39 ± 0.13 (7)		
	5	6.48 ± 0.32 (6)	6.99 ± 0.82 (4)		
Duration of inward movement (Figure 2*D*)	1	1.35 ± 0.22 (4)	3.00 ± 0.27 (5)	1.01 ± 0.17 (5) 60 ± 17% (5)	Shapiro–Wilk W = 0.81, *p* = 0.10 (n.s.) Paired *t* test *t*_(4)_ = 5.820, *p* = 0.002 (sig.) Cohen's d = 2.15
	2	2.83 ± 0.21 (4)	3.55 ± 0.11 (15)		
	3	2.02 ± 0.15 (4)	2.80 ± 0.39 (5)		
	4	1.95 ± 0.21 (4)	2.75 ± 0.23 (9)		
	5	1.56 ± 0.09 (7)	2.68 ± 0.39 (5)		
Duration between inward movements (Figure 2*E*)	1	4.10 ± 0.32 (4)	3.83 ± 0.36 (4)	0.64 ± 0.47 (5) 24 ± 16% (5)	Shapiro–Wilk W = 0.87, *p* = 0.25 (n.s.) Paired *t* test *t*_(4)_ = 1.361, *p* = 0.123 (n.s.) Cohen's d = 0.79
	2	3.15 ± 0.45 (4)	3.80 ± 0.31 (12)		
	3	2.74 ± 0.15 (4)	5.05 ± 0.79 (4)		
	4	2.78 ± 0.33 (3)	3.51 ± 0.15 (7)		
	5	4.89 ± 0.26 (6)	4.66 ± 0.76 (4)		

**Figure 2.**
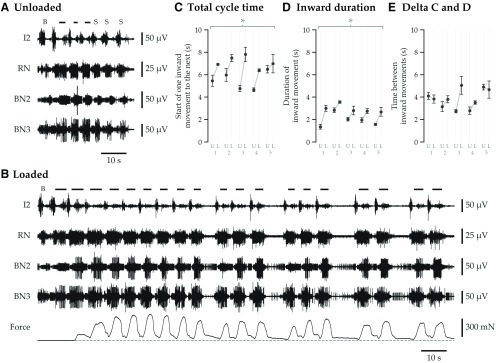
Motor pattern and behavioral differences between unloaded and loaded swallows. Motor patterns were obtained *in vivo* by recording from key components of the motor system: the I2 protractor muscle, whose activation moves the grasper forward; RN, whose largest unit, motor neuron B8a/b, causes closing of the grasper following protraction (Morton and Chiel, 1993b); BN2, whose units can be identified as the motor neurons B3 (largest), B6/B9 (medium), and B38 (medium), which each activate the I1/I3 jaw muscle complex to cause retraction of the grasper (i.e., neurons B3, B6/B9; Lu et al., 2015) and to prevent food from slipping out during protraction (i.e., neuron B38; McManus et al., 2014); and BN3, whose largest unit is the multiaction neurons B4/B5, which serve an important role in the transition between protraction and retraction (Gardner, 1977; Warman and Chiel, 1995). ***A***, Swallows are rapid in the absence of load. This record shows seven complete cycles of ingestive motor programs; with the first, the animal tried to grasp food but failed (bite); with the second, the animal successfully grasped a 5 × 0.5 cm seaweed strip and began swallowing it; by the end of the seventh cycle, the strip was fully consumed, and the animal soon after switched from swallowing to biting (unsuccessful grasping for more food). Above the neural records, “B” indicates the initial bite; bars indicate when the seaweed strip could be clearly seen in the video to be moving inward during a swallow; “S” indicates swallows in which the strip was no longer visible because it had moved completely inside the mouth. The largest unit on BN2, the B3 motor neuron, which is recruited when animals need to generate the greatest force, was activated just twice, during the third motor pattern. Note that the force record is omitted because the seaweed strip was not attached to the force transducer. ***B***, Swallows are slowed and motor recruitment increases in response to external load. Data are obtained from the same animal as in ***A***. At the beginning of the record, the animal was presented with a 10 × 0.25 cm strip of unbreakable tape-reinforced seaweed attached to a force transducer. With the first motor pattern, the animal bit and failed to grasp the strip; with the second, the animal succeeded in grasping and began to swallow; with the third, the strip began to grow taut, and force can be seen (dashed line indicates zero force); by the fifth, the strip was completely taut at peak retraction. The measured force then rose and fell periodically with the retraction phase of each swallow. Because the strip could not break, the animal fed near the surface of the water, with the anterior portion of its head lifting out of the water some short distance with each effort to ingest the strip; as each retraction phase ended, the animal lost its momentary progress up the strip and fell gently back to the surface of the water, with force declining during this time. As in ***A***, video was used to track when seaweed was moving into the mouth, which is indicated with lines above the neural records; the durations of inward movement were initially much longer compared with when external load was completely absent (compare ***A***), and consequently the overall rate of swallowing was slower. Note that although the durations of inward movement were longer, the amplitude of inward movement may not have been very different due to the large mechanical load reducing the speed of inward movement (i.e., the amplitude of inward movement is limited by the amplitude of grasper movement). In contrast to unloaded swallowing, the B3 motor neuron (largest unit on BN2) was usually very active during retraction. After the first nine swallows, the animal introduced pauses between swallowing bouts. After ∼3 min of swallowing without successfully breaking the seaweed, the animal gradually changed from tugging to a radula scraping strategy (data not shown) and eventually released and moved away from the food. For subsequent analyses, only the first, least variable swallows on unbreakable seaweed were used, excluding variation due to changing behavioral strategies, and only swallows after tension in the strip had fully developed were analyzed. ***C–E***, Total cycle time and the duration of inward movement increase in response to load, but the time between inward movements does not. Five animals (numbered 1–5) swallowed unloaded (U) and loaded (L) seaweed strips. Cycles were partitioned by the inward movement of the seaweed strips. The total cycle time (time from start of one inward movement to the next), as well as the period of inward movement, showed statistically significant increases in response to load, whereas the time between inward movements, when the seaweed was either moving outward or was stationary, did not. Mean values are plotted for each animal; whiskers show the SEM. The asterisks above the plots indicate statistically significant increases. See Table 2 for statistical details.

Data are plotted in Figure 2*C–E*. Units are in seconds, and values are reported as the mean ± SEM (sample size). For the total cycle time (Fig. 2*C*) and duration between inward movements (Fig. 2*E*), the numbers of swallows are smaller for some animals compared with the duration of inward movement (Fig. 2*D*) because swallows preceded by another whose inward movement could not be reliably measured were dropped. sig., Significant test results; n.s., nonsignificant test results.

When statistically analyzing differences in muscle and neuronal activity measured in swallows obtained under unloaded and loaded conditions, we divided identified units into the following three groups: protraction phase motor units; retraction phase motor units; and a multiaction unit. The first group was composed of the following two variables: the burst duration of specialized jaw motor neuron B38 (McManus et al., 2014); and the burst duration of the I2 protractor muscle. The second group was also composed of two variables: the burst duration of the grasper closure motor neurons B8a/b; and the burst duration of the combined retractor muscle motor pool of B3 and B6/B9. For these multivariate groups, we deployed paired-samples Hotelling’s *T*^2^ tests; significant test results were followed by *post hoc* paired-samples one-tailed *t* tests on each variable. The third group was in fact univariate: the burst duration of the multiaction B4/B5 neurons. Because this group was univariate, we deployed only a paired-samples one-tailed *t* test. At the group level, where comparisons were planned, *p* < 0.05 was considered significant. For *post hoc* tests on the bivariate groups, a Bonferroni correction was used to control for multiple comparisons, and *p* < 0.05/2 = 0.025 was considered significant. Test results are reported in Table 3, along with effect sizes (Cohen’s d). With mean burst durations taken from five animals, power/sensitivity analyses indicated that the paired-samples Hotelling’s *T*^2^ tests had sufficient sensitivity to detect an effect size of Δ = 2.49 (Faul et al., 2007) with power 1 − β = 0.8 at the α = 0.05 significance level; the group-level paired-samples one-tailed *t* tests conducted on B4/B5 had sufficient sensitivity to detect a standard mean difference of dz = 1.36 with power 1 − β = 0.8 at the α = 0.05 significance level; the *post hoc* paired-samples one-tailed *t* tests had sufficient sensitivity to detect a standard mean difference of dz = 1.68 with power 1 − β = 0.8 at the α = 0.025 significance level.

**Table 3:** Statistical details for changes in burst duration under unloaded and loaded swallowing conditions

Measure (Figure)	Animal	Unloaded	Loaded	Difference	Statistical test results
B38 duration (Figure 4*A*)	1	1.44 ± 0.19 (10)	1.72 ± 0.13 (5)	0.11 ± 0.14 (5) 12 ± 14% (5)	Protraction phase durations Hotelling’s *T*^2^ *T*^2^ = 4.751, *F*_(2,3)_ = 1.781, *p* = 0.309 (n.s.) *Post hoc* tests not conducted
	2	1.92 ± 0.32 (6)	1.81 ± 0.13 (15)		
	3	0.50 ± 0.24 (6)	0.49 ± 0.37 (5)		
	4	0.94 ± 0.19 (6)	1.51 ± 0.15 (9)		
	5	1.45 ± 0.31 (11)	1.24 ± 0.37 (5)		
I2 duration (Figure 4*B*)	1	1.46 ± 0.10 (10)	1.12 ± 0.10 (5)	−0.28 ± 0.13 (5) −18 ± 8% (5)	
	2	1.63 ± 0.15 (6)	1.83 ± 0.21 (15)		
	3	1.61 ± 0.03 (6)	1.36 ± 0.26 (5)		
	4	1.58 ± 0.19 (6)	1.16 ± 0.06 (9)		
	5	1.68 ± 0.13 (11)	1.12 ± 0.18 (5)		
B8a/b duration (Figure 4*C*)	1	3.13 ± 0.22 (10)	3.99 ± 0.17 (5)	0.95 ± 0.21 (5) 32 ± 8% (5)	Retraction phase durations Hotelling’s *T*^2^ *T*^2^ = 59.100, *F*_(2,3)_ = 22.163, *p* = 0.016 (sig.) *Post hoc* test: B8a/b duration Shapiro–Wilk W = 0.99, *p* = 0.97 (n.s.) Paired *t* test *t*_(4)_ = 4.544, *p* = 0.005 (sig.) Cohen's d = 3.13 *Post hoc* test: B3/B6/B9 duration Shapiro–Wilk W = 0.97, *p* = 0.86 (n.s.) Paired *t* test *t*_(4)_ = 6.081, *p* = 0.002 (sig.) Cohen's d = 5.16
	2	3.58 ± 0.22 (6)	4.31 ± 0.15 (15)		
	3	2.73 ± 0.19 (6)	4.31 ± 0.40 (5)		
	4	2.86 ± 0.11 (6)	4.07 ± 0.22 (9)		
	5	3.34 ± 0.24 (11)	3.70 ± 0.51 (5)		
B3/B6/B9 duration (Figure 4*D*)	1	1.87 ± 0.20 (10)	2.77 ± 0.16 (5)	1.12 ± 0.18 (5) 69 ± 16% (5)	
	2	1.66 ± 0.25 (6)	2.71 ± 0.11 (15)		
	3	1.38 ± 0.10 (6)	3.14 ± 0.26 (5)		
	4	1.72 ± 0.09 (6)	2.98 ± 0.11 (9)		
	5	2.01 ± 0.22 (11)	2.67 ± 0.52 (5)		
B4/B5 duration (Figure 4*E*)	1	1.58 ± 0.33 (10)	1.84 ± 0.26 (5)	0.78 ± 0.21 (5) 41 ± 15% (4)	Shapiro–Wilk W = 0.96, *p* = 0.82 (n.s.) Paired *t* test *t*_(4)_ = 3.714, *p* = 0.010 (sig.) Cohen's d = 0.70
	2	2.07 ± 0.47 (6)	3.09 ± 0.33 (15)		
	3	0 ± 0 (6)	0.65 ± 0.41 (5)		
	4	1.79 ± 0.37 (6)	3.25 ± 0.36 (9)		
	5	2.85 ± 0.20 (11)	3.36 ± 0.43 (5)		

Data are plotted in Figure 4*A–E*. Units are in seconds, and values are reported as mean ± SEM (sample size). sig., Significant test results; n.s., nonsignificant test results.

The same series of tests were performed on differences in mean firing frequency during bursting under unloaded and loaded conditions, and results are reported in Table 4.

**Table 4:** Statistical details for changes in mean firing frequency during bursting under unloaded and loaded swallowing conditions

Measure (Figure)	Animal	Unloaded	Loaded	Difference	Statistical test results
B38 frequency (not plotted)	1	10.95 ± 1.80 (10)	10.57 ± 0.70 (5)	0.55 ± 0.57 (5) 3 ± 8% (5)	Protraction phase frequencies Hotelling’s *T*^2^ *T*^2^ = 11.324, *F*_(2,3)_ = 4.247, *p* = 0.133 (n.s.) *Post hoc* tests not conducted
	2	10.44 ± 0.84 (6)	11.40 ± 0.33 (15)		
	3	4.50 ± 2.02 (6)	3.35 ± 2.06 (5)		
	4	11.88 ± 2.85 (6)	13.57 ± 0.37 (9)		
	5	7.41 ± 1.49 (11)	9.04 ± 0.93 (5)		
I2 frequency (not plotted)	1	15.17 ± 0.42 (10)	15.71 ± 0.92 (5)	1.21 ± 0.49 (5) 9 ± 4% (5)	
	2	12.33 ± 0.85 (6)	12.43 ± 0.62 (15)		
	3	12.80 ± 0.44 (6)	15.66 ± 0.70 (5)		
	4	15.37 ± 0.37 (6)	16.15 ± 0.81 (9)		
	5	12.92 ± 0.37 (11)	14.68 ± 0.78 (5)		
B8a/b frequency (not plotted)	1	12.00 ± 0.87 (10)	12.20 ± 0.61 (5)	1.59 ± 0.93 (5) 10 ± 5% (5)	Retraction phase frequencies Hotelling’s *T*^2^ *T*^2^ = 29.446, *F*_(2,3)_ = 11.042, *p* = 0.041 (sig.) *Post hoc* test: B8a/b frequency Shapiro–Wilk, W = 0.95, *p* = 0.75 (n.s.) Paired *t* test, *t*_(4)_ = 1.710, *p* = 0.081 (n.s.) Cohen's d = 0.28 *Post hoc* test: B3/B6/B9 frequency Shapiro–Wilk, W = 0.96, *p* = 0.84 (n.s.) Paired *t* test, *t*_(4)_ = 3.935, *p* = 0.009 (sig.) Cohen's d = 0.47
	2	18.22 ± 1.27 (6)	22.91 ± 0.93 (15)		
	3	21.61 ± 2.79 (6)	23.40 ± 2.22 (5)		
	4	22.20 ± 1.04 (6)	21.46 ± 1.72 (9)		
	5	10.32 ± 0.51 (11)	12.32 ± 0.72 (5)		
B3/B6/B9 frequency (Figure 4*F*)	1	38.99 ± 2.23 (10)	42.27 ± 1.81 (5)	5.82 ± 1.48 (5) 31 ± 11% (5)	
	2	14.79 ± 0.94 (6)	16.59 ± 1.02 (15)		
	3	27.85 ± 1.46 (6)	37.90 ± 1.43 (5)		
	4	30.40 ± 2.24 (6)	37.87 ± 1.03 (9)		
	5	9.12 ± 0.72 (11)	15.66 ± 1.04 (5)		
B4/B5 frequency (not plotted)	1	21.29 ± 1.59 (10)	17.57 ± 1.14 (5)	1.75 ± 1.41 (5) 17 ± 11% (4)	Shapiro–Wilk W = 0.74, *p* = 0.025 (sig.) Paired Wilcoxon signed-rank W = 11, *p* = 0.22 (n.s.) Cohen's d = 0.25
	2	7.43 ± 1.62 (6)	9.27 ± 0.97 (15)		
	3	0 ± 0 (6)	3.44 ± 2.66 (5)		
	4	10.47 ± 1.24 (6)	13.81 ± 1.00 (9)		
	5	13.97 ± 0.49 (11)	17.85 ± 0.97 (5)		

Data for B3/B6/B9 are plotted in Figure 4*F* (changes for other units were not significant). Units are in hertz, and values are reported as the mean ± SEM (sample size). sig., Significant test results; n.s., nonsignificant test results.

Shapiro–Wilk tests for deviations from normality (Sheskin, 2011) were also performed before every *t* test (Tables 3, 4) to determine whether the assumption of normality of the *t* test was violated. If the results were not significant (which occurred in all but one case), suggesting the assumption was not violated, we proceeded with a *t* test. Otherwise, we substituted a paired-samples one-tailed Wilcoxon signed-rank test, which had slightly less sensitivity than a *t* test (dz = 1.41 with power 1 − β = 0.8 at the α = 0.05 significance level; dz = 1.76 with power 1 − β = 0.8 at the α = 0.025 significance level).

For comparing motor neuronal duration and force duration, we report in the figure legend the coefficients of determination (*R*^2^) and *p* values obtained from linear regression. *p* < 0.05 was considered significant. Sample sizes were small in some cases, but because we found strong correlations in every case, the risk of false-negative results was not too large. Achieved power was 0.95 for all animals combined, and 0.29, 0.61, 0.31, 0.54, and 0.32 for the five animals individually at the α = 0.05 significance level (Faul et al., 2007).

Hotelling’s *T*^2^ tests, Shapiro–Wilk tests, *t* tests, Wilcoxon signed-rank tests, and Cohen’s d calculations were performed in R, linear regression was performed in Python using the StatsModels package (www.statsmodels.org), and power analyses were performed using G*Power (Faul et al., 2007). Sheskin (2011) was consulted for statistical procedures. Experiments were not preregistered.

#### Code and data availability

Analyses were performed using custom Python code that made use of the neurotic (Gill et al., 2020) and Neo (Garcia et al., 2014) packages. The code is availableat GitHub (https://github.com/CWRUChielLab/Gill-Chiel-eNeuro-2020-code). The code automates retrieval of data files and reproduces figures and statistical analyses. Data are archived at GIN (Gill and Chiel, 2020).

## Results

### Behavioral changes in response to load

Previous studies have shown that animals consistently alter their behavior in response to increasing load (Hurwitz and Susswein, 1992). These studies were performed in *A. oculifera* (on average, 7.4 g in size). We first sought to confirm that similar behavioral observations could be made in *A. californica*, which is a much larger species (200–450 g in this study). To evoke feeding behavior under unloaded and loaded conditions, each animal was presented with the following two stimuli at different times (see Materials and Methods): unloaded seaweed strips and unbreakable seaweed strips anchored to a force transducer.

Animals readily ingested unloaded 5 cm strips in three to five swallows. Figure 2*A* shows a typical sequence of motor patterns generated before, during, and after this behavior. The frequency of firing and duration of bursts of many retraction phase neurons increased after the animal successfully grasped food, remained elevated throughout swallowing, and finally decreased once the strip was fully ingested.

In contrast, when feeding on unbreakable, anchored strips (Fig. 1*B*,*C*, example video frames), animals could not finish ingesting the strips, so they generated many more swallowing motor patterns, sometimes for several minutes. Once the strip was taut, animals rhythmically generated tension on it with each swallow, but ceased to be able to effectively pull food into the mouth. Figure 2*B* shows the start of such a sequence of motor patterns, along with force. Compared with unloaded swallows, the increases in firing frequency and burst duration of retraction phase neurons in loaded swallows when animals shifted from biting to swallowing can be seen to be even greater. Furthermore, the B3 motor neuron (largest unit on BN2 because it has the largest soma and axon), which activates to greatly increase retraction force (Lu et al., 2015), was recruited much more frequently.

Swallowing was slowed when animals engaged with load (Table 2, all statistical details). Bars at the top of Figure 2, *A* and *B*, show the periods during which the animal was moving food into the mouth. Using this behavioral marker, across several animals and many swallows, the time from one cycle to the next increased significantly in the presence of load (mean ± SEM increase: 1.65 ± 0.41 s, or 32 ± 9%; Fig. 2*C*). This is consistent with prior findings by Hurwitz and Susswein (1992). Is the increase in duration uniform across all phases of swallowing? The amount of time the animal spent pulling food in when load was present, which corresponds to the retraction phase of swallowing, was significantly increased (mean ± SEM increase: 1.01 ± 0.17 s, or 60 ± 17%; Fig. 2*D*), but the amount of time between the inward movements, which corresponds to the protraction phase of swallowing, was not (mean ± SEM increase: 0.64 ± 0.47 s, or 24 ± 16%; Fig. 2*E*).

### How individual neuronal activity shapes behavior

**Figure 3.**
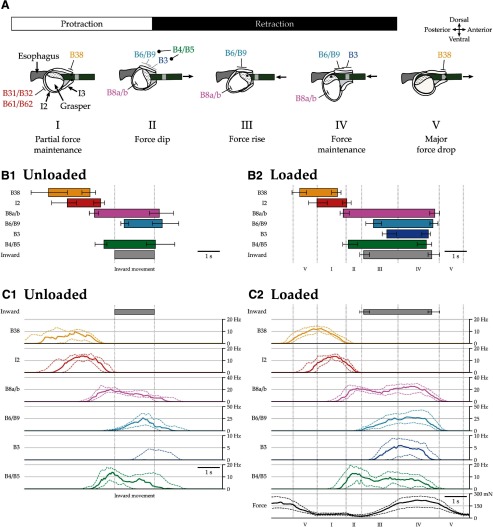
Biomechanics, timing, and frequency of motor neuronal activity during unloaded versus loaded swallowing. A schematic of the biomechanics of swallowing helps explain the changes in the timing and frequency of motor neuronal activity between unloaded and loaded swallows. ***A***, The biomechanics and motor control of swallowing. The stages of swallowing a seaweed strip under tension are illustrated schematically in a midsagittal view of the buccal mass, with the anterior opening of the mouth at the right and the esophagus at the left. Panels correspond to the five phases observed in the force record. See Materials and Methods for force segmentation procedure, and see Results for a detailed explanation of each stage. Closing of the grasper is illustrated by a change of shape from roughly spherical (stages I, II, and V) to ellipsoidal (stages III and IV; Neustadter et al., 2002). Points of contact between the seaweed and the buccal mass are indicated by black dots. The schematic in ***A*** was modified from Cullins et al. (2015b). ***B1–C2***, Muscle and identified neuronal activity during unloaded and loaded swallowing. ***B1***, ***B2***, The timing of bursts of identified motor units are plotted for swallows from five animals on unloaded seaweed strips (left; *n* = 4, 4, 4, 4, 7 swallows) and on anchored, unbreakable seaweed strips (right; *n* = 5, 15, 5, 9, 5 swallows). Boxes indicate median timing, and whiskers indicate the lower and upper quartiles for the beginnings and endings of bursts. The period of seaweed inward movement is similarly indicated. ***C1***, ***C2***, The firing frequencies of the units are plotted for the same datasets. Thick lines indicate median frequencies, and dashed lines indicate the lower and upper quartiles for frequency. For loaded swallows (***C2***), force is similarly plotted, and the drop in force at the end of the previous swallow can be seen at the start (initial stage V). To aggregate swallows, time was normalized using different methods for unloaded and loaded swallows that still permit comparison (see Materials and Methods). Vertical dotted lines indicate the boundaries of segmentation used for normalization. For unloaded swallows, behaviors were aggregated by normalizing time using the period of inward seaweed movement; inward movement timing was therefore invariant after normalization, so whiskers are omitted in the left panels. For loaded swallows, behaviors were aggregated by normalizing time using segmentation of the force record; inward movement was therefore variable after normalization, so whiskers are shown in the right panels. For unloaded swallows, the B3 retractor motor neuron was recruited so rarely that it burst in only 6 of 23 swallows; consequently its typical burst timing (***B1***) is not plotted, and its typical firing frequency (***C1***) was so low that only the upper quartile is visible. Overall, the durations of bursts of the B8a/b closure motor neurons, the combined B3/B6/B9 retractor motor neurons, and the B4/B5 multiaction neurons were longer under load; in contrast, the burst durations of protraction phase units B38 and I2 were not significantly different (Fig. 4, quantification). Likewise, the intensities of firing of the B6/B9 and B3 motor neurons during retraction were greater in the presence of load (Fig. 4, quantification).

Understanding how the activity of identified motor neurons leads to functionally significant behavioral changes requires an understanding of the biomechanics and motor control of swallowing. The mouth is composed of a muscular organ known as the buccal mass (feeding apparatus). The movement of food through the buccal mass is primarily performed by the grasper (whose surface is the radula, and whose underlying musculature and cartilage are referred to as the odontophore), which closes to grasp food and opens to release it; the grasper also pivots on its base (Howells, 1942; Neustadter et al., 2007). Feeding motor programs have the following two major phases: protraction of the grasper, when it pivots anteriorly, and retraction of the grasper, when it pivots posteriorly. The multifunctional feeding apparatus can perform both ingestive behaviors (biting and swallowing) and egestive behaviors (rejection of unpalatable or inedible food; Kupfermann, 1974; Neustadter et al., 2007). The difference depends on the timing of closing of the grasper: in ingestive behaviors, the grasper is closed during retraction to pull food in, whereas in egestive behaviors, the grasper is closed during protraction to push inedible food out (Morton and Chiel, 1993a). The kinematics of swallowing are illustrated schematically in Figure 3*A*.

**Figure 4.**
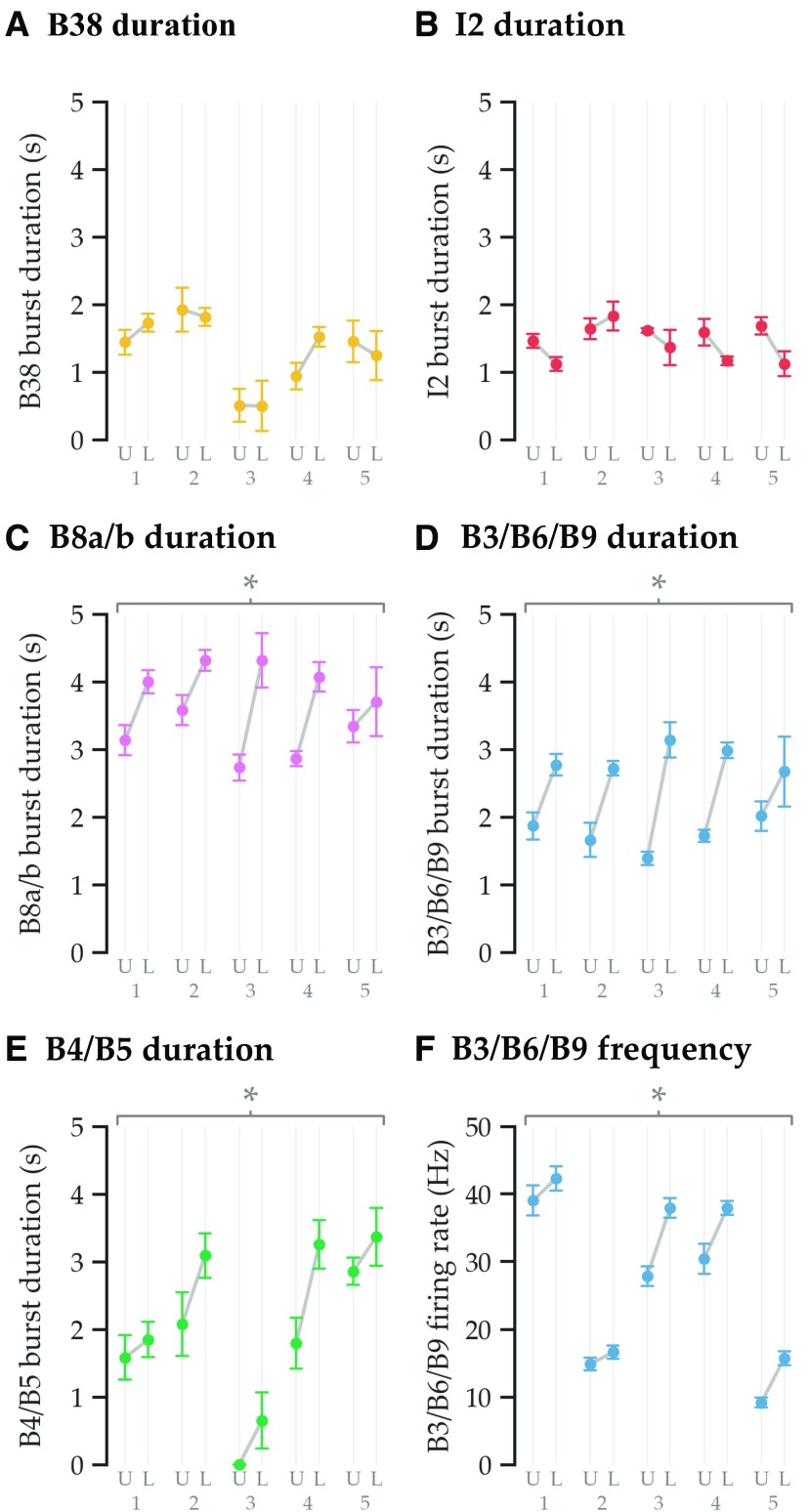
Changes in burst duration and mean firing rate associated with load. ***A***, ***B***, Protraction phase motor neuron activity (B38 and I2) was unchanged by load. ***C***, ***D***, ***E***, In contrast, the durations of retraction phase motor neuron activity (B8a/b and B3/B6/B9) and B4/B5 activity increased when load was present. ***F***, The firing frequency of retractor muscle motor neurons (B3/B6/B9) also increased when load was present. Mean values are plotted for each animal; whiskers show the SEM. The asterisks above the plots indicate statistically significant increases in duration and firing rate. For statistical details, see Tables 3 and 4. Numbers 1-5 indicate individual animals. U, unloaded; L, loaded.

Force records obtained while animals swallowed loaded seaweed strips provide a detailed window into the stages of swallowing and offer additional insight for interpreting changes in neuronal activity. We observed a regular pattern in the force record (Fig. 2*B*, example) that suggested force generation could be divided into behaviorally relevant subphases. Using the boundaries shown in Figure 1*A*, we segmented swallows into five stages, whose kinematics are illustrated in Figure 3*A*.

#### Stage I: partial force maintenance

The first stage of the swallowing cycle corresponds to protraction of the grasper (Fig. 3*A*, stage I), when the protractor muscle I2 is activated by the B31/B32 and B61/B62 motor neurons (Hurwitz et al., 1996). This stage follows the end of retraction in the previous swallow, when the force on the seaweed drops very rapidly to an intermediate value (Fig. 1*A*, stage I), and ends with the peak of protraction. The rapid reduction in force preceding this stage is due to the opening of the grasper and the cessation of retraction. During this stage, in many but not all swallows, the force is partially maintained at an intermediate level even as the open grasper protracts. This is accomplished by the specialized B38 motor neuron, which selectively activates the anterior portion of the jaws to clamp down on the food anterior to the grasper (McManus et al., 2014), which might otherwise be lost due to the food being under tension.

#### Stage II: force dip

The second stage of the force segmentation occurs at the peak of protraction (Fig. 3*A*, stage II). The grasper protracts sufficiently far that, in swallows with partial force maintenance in stage I, the anterior portion of the jaws is forced open and contact between the jaws and the seaweed is lost; alternatively, the activity of the B38 motor neuron may cease and allow the jaws to relax, leading to the same result. Consequently, the force drops again to a new minimum value (Fig. 1*A*, stage II). During this stage, the I2 protractor muscle ceases to push the grasper anteriorly, and the grasper closing motor neurons B8a/b begin to fire, inducing the grasper to begin closing (Morton and Chiel, 1993b). At the same time, the multiaction neurons B4/B5 begin firing, which often signals the border between protraction and retraction (Warman and Chiel, 1995). B4/B5 inhibit the retractor motor neurons B6/B9 and B3 (Gardner, 1977), delaying the onset of their activity.

#### Stage III: force rise

The third stage of the force segmentation corresponds approximately to the first half of retraction, when the grasper is closed and begins to move posteriorly (Fig. 3*A*, stage III). This is the onset of the power stroke of swallowing, when force begins to increase rapidly and the food moves inward (Fig. 1*A*, stage III). The grasper closes and remains closed through the continued firing of B8a/b. Shortly after the onset of B8a/b firing, retractor motor neurons B6/B9, and somewhat later the retractor motor neuron B3, fire intensely, causing contraction of the I3 jaw muscle (Church and Lloyd, 1994; Lu et al., 2015). This pushes the grasper backward, causing a rapid increase in force (Lu et al., 2015).

#### Stage IV: force maintenance

The fourth stage of the force segmentation corresponds to the second half of retraction, during which the grasper reaches a fully retracted position and food ceases to move inward (Fig. 3*A*, stage IV). During this stage, force reaches its peak value and is maintained at this high level (Fig. 1*A*, stage IV) by the continued intense firing of B6/B9 and B3 (Lu et al., 2015).

#### Stage V: major force drop

The fifth stage of the force segmentation occurs just after the end of retraction (Fig. 3*A*, stage V). At this time, the force rapidly drops (Fig. 1*A*, stage V), and the seaweed, which is under tension, may slide partially out beyond the jaws. This is caused by the cessation of firing of B8a/b, which allows the grasper to open, and the cessation of firing of B6/B9 and B3, which allows the I3 jaw muscle to partially relax, permitting the grasper to start returning to a neutral position. The B38 motor neuron may begin to fire at this time, selectively maintaining and intensifying contraction of the anterior portion of the jaws, which reduces the amount of food lost (McManus et al., 2014). In some swallows, this is sufficient to partially maintain some of the force generated in stages III and IV. The cycle then repeats.

Overall, although there was variability between and within animals, the timing of neuronal activity relative to events in the force record was fairly consistent, and is summarized in Figure 3*B2*. B38 activity, when present, began in stage V of the previous swallow and continued into stage I; activity of the I2 protractor muscle reliably occurred during stage I; onset of B8a/b and B4/B5 activity began at the start of stage II; B6/B9 activity began during stage III after B8a/b and onset of B3 activity, when present, usually followed B6/B9; and B8a/b, B6/B9, and B3 all ceased firing at the end of stage IV.

Animals sometimes interjected long pauses between swallows during which the motor neurons were relatively quiescent and the muscles were relaxed (Fig. 2*B*, examples). For our analysis, we only used swallows thatpreceded the first pause, but these pauses likely corresponded to an extended stage I (partial force maintenance), during which most force was lost.

### Adaptive changes in neuronal activity in response to load

To see how the activities of identified neurons change in the presence of load across multiple responses and multiple animals, we normalized time so that swallows could be combined (see Materials and Methods). Loaded swallows were normalized using the force segmentation previously described; unloaded swallows were normalized using the video record of inward seaweed movement because a force record was unavailable.

Durations of firing of identified retractor motor neurons are increased in response to load (Table 3, all statistical details). Figure 3, *B1* and *B2*, shows the median timing and durations of bursts and of inward seaweed movement in unloaded and loaded swallows, respectively. Notably, the durations of activity of the motor units active in protraction—B38 and I2—are very similar regardless of whether load is present or not. When the durations of these units are compared within animals without time normalization (Fig. 4*A*,*B*), there are no significant changes associated with load (mean increase ± SEM: B38, 0.11 ± 0.14 s, or 12 ± 14%; I2, −0.28 ± 0.13 s, or −18 ± 8%). This is consistent with the result that the time between inward movements of the food is unaffected by load (Fig. 2*E*). (In a previous study in which animals were presented with inedible food (McManus et al., 2019), the duration of protraction was increased, but this was because the inedible food was pressed against the feeding apparatus to ensure the animal) retained it.) In contrast, the motor units active in retraction—B8a/b, B6/B9, and B3—which are responsible for closing and retracting the grasper, burst for longer when load is present, just as the inward movement duration was increased by load (Fig. 2*D*). B3 burst when animals fed on unloaded seaweed in only 6 of 23 swallows, so the bar corresponding to B3 is omitted in Figure 3*B1*. When these durations are compared within animals without time normalization (Fig. 4*C*,*D*; B3 was grouped with B6/B9 because of their functional similarity and because B3 is usually inactive when load is absent), each increases significantly with load (mean increase ± SEM: B8a/b, 0.95 ± 0.21 s, or 32 ± 8%; B3/B6/B9, 1.12 ± 0.18 s, or 69 ± 16%). Likewise, the retraction phase multiaction neurons B4/B5 burst for longer when load is present, and this effect persists when animals are used as their own control without time normalization (mean increase ± SEM: 0.78 ± 0.21 s, or 41 ± 15%; Fig. 4*E*).

Frequencies of firing of identified retractor motor neurons are increased in response to load (Table 4, all statistical details). Figure 3, *C1* and *C2*, shows the median firing rates of each unit in unloaded and loaded swallowing, respectively, as well as the median force for loaded swallows. The activity of each unit before the start of inward seaweed movement—when load would have little effect on the grasper because it is open—depends very little on the feeding stimulus. After the start of inward seaweed movement—when load could affect the closed grasper—the activity of each retraction phase neuron deviates quickly when load is added. The firing rates of closure motor neurons B8a/b and retractor motor neurons B6/B9 rise to new maxima, and the B3 retractor motor neuron is strongly recruited. Together, these extend the retraction phase and increase the force generated by the power stroke of swallowing. In particular, the recruitment of B3, which has the largest soma, axon, and effect on force among neurons in the retractor motor pool (Lu et al., 2015, their Fig. 7), and the increased frequency of B6/B9 are consistent with Henneman’s size principle, previously described in vertebrate systems (Henneman et al., 1965; Mendell, 2005; Binder et al., 2011). The multiaction neurons B4/B5 also have an extended period of activity (though their peak firing rate still occurs at the protraction–retraction boundary, when they may act to delay the onset of activity of the retractor motor neurons B3/B6/B9). In particular, the mean firing rate of retractor motor neurons B3/B6/B9 increases in the presence of load (mean increase ± SEM: 5.82 ± 1.48 Hz, or 31 ± 11%; Fig. 4*F*).

### Variability in force duration associated with motor neuron activity

In nature, animals can break off pieces of seaweed they are attempting to swallow by pulling for longer and with greater force. We have already seen that animals activate the major retractor motor neurons B3 and B6/B9 forlonger when encountering load (Fig. 4*D*). How does increasing the duration of motor neuronal activity relate to force duration?

**Figure 5.**
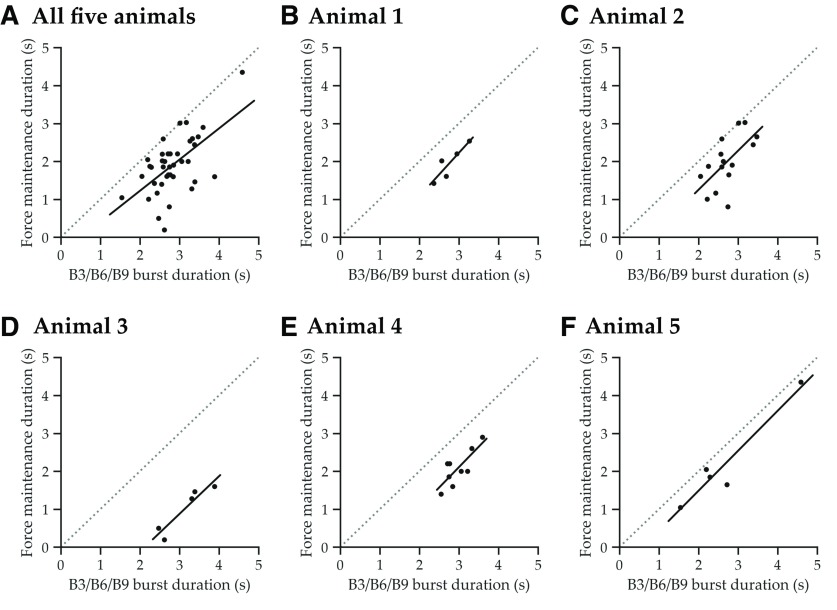
Duration of retractor motor neuronal activity predicts the duration of high force. For individual swallows on loaded unbreakable seaweed strips, the duration of bursting of the B3/B6/B9 retractor motor neurons is plotted against the duration of the force maintenance phase (stage IV; Fig. 3). Dotted lines indicate the 45º angle line, where a 1:1 temporal relationship between motor neuronal activity and force would be found; points are all below these lines because the force maintenance phase does not include the start of the motor neuronal activity. ***A***, When swallows from all animals are grouped together, there is a statistically significant overall linear relationship between the duration of retractor motor neuronal activity and force maintenance (*R*^2^ = 0.36, *p* = 0.00006, *n* = 39). ***B*–*F***, When animals are considered separately, the relationship is stronger because animals may occupy different regions of the space (animal 1, *R*^2^ = 0.81, *p* = 0.038, *n* = 5; animal 2, *R*^2^ = 0.39, *p* = 0.013, *n* = 15; animal 3, *R*^2^ = 0.88, *p* = 0.019, *n* = 5; animal 4, *R*^2^ = 0.63, *p* = 0.011, *n* = 9; animal 5, *R*^2^ = 0.92, *p* = 0.010, *n* = 5).

Force maintenance is highly correlated with the duration of activity in retractor motor neurons B3/B6/B9 (Fig. 5, statistical details in legend). Figure 5*A* plots, for individual swallows on loaded, unbreakable seaweed from five animals, the duration of force maintenance (stage IV) versus the duration of bursting of B3/B6/B9 (these neurons are grouped together because of their functional similarity). When all five animals are plotted together, there is a significant linear relationship between force maintenance duration and motor neuronal burst duration (Fig. 5*A*). Because of variability between animals, that linear relationship is even stronger when animals are plotted separately (Fig. 5*B–F*). Some animals occupy nonoverlapping regions of the space(e.g., animals 3 and 5), so the variability within these animals can be better understood by considering them as individuals.

## Discussion

To our knowledge, this is the first characterization of size-ordered recruitment of identified motor neurons in intact behaving *Aplysia* in response to increased mechanical loading. Load slows swallowing (Fig. 2), consistent with results reported by Hurwitz and Susswein (1992), and primarily acts to extend the retraction phase (Fig. 2*D*). Across animals whose neural recordings are normalized by behavioral durations, load selectively changes the neurons associated with the retraction phase: closure motor neurons B8a/b, retractor motor neurons B3 and B6/B9, and the multiaction neurons B4/B5 (Fig. 3). When animals are used as their own controls without time normalization, increases in duration are seen in the B8a/b, B3, and B6/B9 motor neurons, as well as the B4/B5 multiaction neurons in response to increased load (Fig. 4). The mean firing frequency of the B3/B6/B9 motor neurons also increased in response to mechanical load (Fig. 4*F*). The duration of force maintenance is correlated to the duration of activity of the B3/B6/B9 neurons, which are responsible for the retraction power stroke during swallowing (Fig. 5). The B3 motor neuron, which has the largest axon and the largest effect on contracting the I1/I3 jaw muscle (Lu et al., 2015, see their Fig. 7), is recruited most strongly in response to large loads. The recruitment of the B3 neuron and the increase in the firing rate of B6/B9 are consistent with Henneman’s size principle in vertebrates: smaller-diameter motor axons are recruited before larger-diameter motor axons (Henneman et al., 1965; Mendell, 2005; Binder et al., 2011). Given that the I1/I3 muscle motor pool is very small, it is reasonable that animals would recruit relatively few neurons to increase force output. Thus, animals adapt neuronal activity and behavior on a moment-to-moment basis.

We confirm observations by Hurwitz and Susswein (1992) that swallowing is slowed by increasing mechanical load, and the findings of Lum et al. (2005), who concluded that variability in neuronal activity is reflected in behavioral variability. However, Lum et al. (2005) also concluded, from experiments with small changes in load, that “functional performance is not determined strongly by one or a few parameters of the internal activity,but weakly by many.” In contrast, when motor task requirements change because of large increases in load, we find that it is possible to directly relate neuronal activity to behavior, provided that the biomechanical context and the functional role of individual neurons are understood. In general, knowledge of motor neuronal activity alone may be insufficient to predict behavior without a deeper understanding of the biomechanical context (Hooper and Weaver, 2000).

Although the present study has limitations, they do not weaken its conclusions. We did not test the responses of animals to natural seaweeds, but used uniform seaweed strips. This eliminated one source of variability that could have made the data difficult to interpret, and made it easier to compare stimuli with and without high load. Preliminary studies using natural seaweed attached to a force transducer showed similar force profiles before the seaweed broke. Although force contributions from other parts of the body were not explicitly eliminated, correlates of force generation were still identified in the neural responses. Obtaining behavioral and neuronal responses in unrestrained animals ensured that animals produced more natural responses.

The high variability observed by Lum et al. (2005) made it difficult to relate neural correlates to behavior. The relationship between neural activity and behavior was clarified by using biomechanics to guide our analysis. In particular, by using the behavior (video alone or video and force) to segment unloaded and loaded swallows (respectively), biomechanically relevant epochs were defined that determined behaviorally meaningful neural correlates.

Although we have shown an important role for motor neurons B3/B6/B9 during force maintenance, other motor neurons may also contribute. Force usually rose (stage III) before the major retractor motor neurons B6/B9 and B3 began to fire intensely (Fig. 3*B2*). Some of the initial force may be generated by passive hinge forces (Sutton et al., 2004a,b), and some by the grasper closing due to B8a/b activity, which in type B (large amplitude) swallows can generate retraction movements (Ye et al., 2006). Motor neuron B7, which projects onto buccal nerve 3 as its third largest unit and innervates the “hinge” at the base of the grasper (Ye et al., 2006), may contribute to this initial force. Like B8a/b, B7 can generate retraction forces in type B (large-amplitude) swallows (Sutton et al., 2004a; Ye et al., 2006). The B15 and B16 motor neurons, which innervate a muscle intrinsic to the grasper called the accessory radular closer (or I5; Cohen et al., 1978; Cropper et al., 1990), and which also project through BN3 (Church and Lloyd, 1991), could also contribute. Other motor neurons that activate the retractor muscle, such as B10 and B39 (Church and Lloyd, 1994), could also contribute to the initial force. Because it is difficult to accurately identify these neurons from nerve recordings alone, we did not include them in our analysis.

### Variability can enhance behavioral efficacy

The biomechanical problems animals must solve are highly variable, so it is unsurprising that behavior and its neural control would be variable. In their natural environment, *Aplysia* feed on a variety of seaweeds with different biomechanical properties, including variation in toughness, texture, and size (Howells, 1942; Susswein et al., 1984; Carefoot, 1987). The biomechanical properties of seaweeds vary significantly to resist breakage (Koehl, 1986) and change biomechanically in response to herbivory (Burnett and Koehl, 2019). Depending on their success, animals may choose to cut seaweed or to tear off a piece when encountering load (Hurwitz and Susswein, 1992). Furthermore, as animals ingest seaweed, it may bunch up in the buccal cavity, changing the internal load with which the animal must deal. Thus, the problem changes even as the animal attempts to solve it.

In the short term, animals incorporate sensory feedback to adapt their behavior on a moment-to-moment, phase-dependent basis (Pearson, 2000). As we have shown, animals may extend the duration of the power stroke (retraction) of swallowing when load is encountered. This is consistent with predictions made by a nominal neuromechanical model of *Aplysia* feeding (Shaw et al., 2015; Lyttle et al., 2017). Similarly, in vertebrate and stick insect locomotion, increased load during the stance phase causes increased excitation to the leg extensor muscles (Pearson, 1995). More generally, sensory feedback may allow animals to perform behaviors in a common solution space, such that motor control may vary within animals yet lead to similar behavioral performance among animals (Cullins et al., 2015a).

In the medium term, state-dependent changes caused by changes in arousal, motivation, and shifting goals may also lead to behavioral modifications or switching (Pearson, 2000). In feeding on loaded, unbreakable seaweed strips, we have observed that after animals have attempted to ingest the food for a few minutes, they may switch to alternative behavioral modes, such as rasping, and ultimately leave the stimulus in search of other food. When encountering noxious stimuli, *Aplysia* may also switch from ingestion to egestion (Kupfermann, 1974; Morton and Chiel, 1993a). More generally, animals exhibit many such changes in behavior in locomotion, feeding, and respiration (Pearson, 2000).

In the long-term, animals may learn to respond differently to challenging motor tasks so as to increase fitness. For example, *Aplysia* may learn that a specific food item is inedible and spend less time attempting to feed on it (Susswein et al., 1986; Chiel and Susswein, 1993; Tam et al., 2020). More generally, animals will associate different foods with different levels of reward and seek out their preferred foods (Petrovich, 2018). Many of the medium-term and long-term behavioral adaptations that animals exhibit are forms of flexibility, through which they remain robust to environmental variation and maintain high fitness (Lyttle et al., 2017).

Previous studies in other systems have identified the behavioral roles of single units in intact animals, such as leg motor neurons in stick insects (Cruse and Pflüger, 1981), the stomatogastric nervous system of crayfish (Böhm et al., 2001), and in the mollusc *Lymnaea stagnalis* (Yeoman et al., 1994). Recently, genetically identified populations of leg motor neurons have been controlled in behaving *Drosophila* using optogenetic techniques and have been shown to follow Henneman’s size principle (Azevedo et al., 2019). An advantage of the feeding system of *Aplysia* is that individual neurons are identifiable and electrically compact, allowing them to be manipulated in semi-intact preparations, making it easier to work out the detailed neural circuitry for control. Many of the key interneurons that activate feeding behaviors and that control switches among them have been identified (e.g., the cerebral–buccal interneurons; Jing and Weiss, 2001, 2005). Semi-intact preparations have been developed that generate different feeding responses (McManus et al., 2012) in which identified neurons can be monitored and manipulated (Lu et al., 2013). Indeed, a recent study demonstrated forms of associative learning using a semi-intact preparation (McManus et al., 2019).

### A framework for motor control

A variety of frameworks has been suggested for understanding motor control. At one extreme, Lum et al. (2005) have suggested that variability may be a trial-and-error process for finding successful behaviors. At the other, McNamee and Wolpert (2019) have proposed that animals may develop internal computational models of the biomechanics governing their own bodies and the environment so as to generate optimal behavior. The results of this study suggest a different framework in which behavior is constantly shaped by sensory feedback, and animals use this to find a “good enough” solution to most problems (Loeb, 2012). This is made possible by the great range of nearly identical adaptive responses available to solve a motor problem (Ting et al., 2015). In addition, biomechanical constraints and affordances help shape behavior (Ting et al., 2015). Ongoing feedback from the environment and the periphery acts to continuously shape and modify motor outputs, and the biomechanics of the periphery are an important part of the solution. Consequently, to understand motor output from the nervous system, it is essential to understand the biomechanical context.

## Synthesis

Reviewing Editor: Trevor Drew, University of Montreal Decisions are customarily a result of the Reviewing Editor and the peer reviewers coming together and discussing their recommendations until a consensus is reached. When revisions are invited, a fact-based synthesis statement explaining their decision and outlining what is needed to prepare a revision will be listed below. The following reviewer(s) agreed to reveal their identity: Mark Miller. Note: If this manuscript was transferred from JNeurosci and a decision was made to accept the manuscript without peer review, a brief statement to this effect will instead be what is listed below.

na
